# Relationship between Pulsatility Index and Clinical Course of Acute Ischemic Stroke after Thrombolytic Treatment

**DOI:** 10.1155/2013/265171

**Published:** 2013-07-25

**Authors:** Nevzat Uzuner, Özcan Özdemir, Gülnur Tekgöl Uzuner

**Affiliations:** Department of Neurology and Department of Cerebrovascular Disease and Stroke Unit, Faculty of Medicine, Eskisehir Osmangazi University, Meselik, 26480 Eskisehir, Turkey

## Abstract

*Background*. The relationship between the arterial recanalization after intravenous recombinant tissue plasminogen activator (rtPA) and outcomes is still uncertain. The aim of our study was to evaluate whether there is an association between the pulsatility indexes (PI) of the middle cerebral artery (MCA) measured by transcranial Doppler (TCD) after iv rtPA treatment and short- and long-term outcomes in ischemic stroke patients. *Methods*. Forty-eight patients with acute ischemia in the MCA territory who achieved complete recanalization after the administration of intravenous thrombolytic treatment were included in the study. The TCD was applied to patients after the iv rtPA treatment. Clinical and functional outcomes were assessed by National Institutes of Health Stroke Scale (NIHSS) scores and modified Rankin Scores (mRS), respectively. *Results*. Significant positive correlations were found between the PI value and NIHSS score at 24 hours, NIHSS score at 3 months, and mRS at 3 months (*P* < 0.005 for all). The cut-off value for PI in predicting a favorable prognosis and a good prognosis might be less than or equal to 1.1 and less than or equal to 1.4, respectively. *Conclusions*. PI may play a role in predicting the functional and clinical outcome after thrombolytic therapy in acute ischemic stroke patients.

## 1. Introduction

Cerebrovascular disease (CVD) is the second leading cause of death and ranks first in terms of disability. It is also an important economic and social health problem. Thrombolysis with intravenous rtPA has been shown to be effective in patients with acute ischemic stroke within 4.5 hours from symptom onset [1]. 

TCD is a noninvasive method that informs about blood flow velocities in the main intracranial arteries and flow directions according to an ultrasound probe [2]. It is suggested that it is a good measure for showing the results of occlusion detected in MCA. TCD can predict clinical response to thrombolysis by detecting the site of vessel occlusion and recanalization rate [3].

The purpose of our study is to evaluate whether PI measured by TCD in the acute stages of MCA territory ischemic stroke in patients with successful recanalization after iv rtPA treatment is a predictor of the course of the disease in the acute and chronic phases.

## 2. Materials and Methods

Patients with acute ischemia in the MCA territory who presented within 4.5 hours after the symptom onset and who received intravenous thrombolytic therapy at Eskisehir Osmangazi University Emergency and the Neurology Department were included in the study from December 2009 to March 2012. Consecutive patients were treated with intravenous tissue plasminogen activator (iv-rtPA) following SITS-MOST criteria [4]. Written confirmation from the Eskisehir Clinical Research Ethics Committee, dated 16.12.2009 and numbered 2009/29, was received for this study and an “Informed Consent Form” was received from all patients or their relatives. 

Patients with insufficient temporal window were excluded from the study. Patients with extracranial carotid stenosis were also excluded, given the possibility of hemodynamic changes in middle cerebral artery blood flow velocities due to proximal stenosis. 

All patients were admitted to our Neurocritical Care Unit. A detailed past medical history was obtained from each patient. To identify the potential mechanism of stroke, patients underwent transthoracic and transesophageal echocardiography, Holter monitoring, and magnetic resonance angiography when indicated. The Trial of Org 10172 in Acute Stroke Treatment Criteria (TOAST) criteria was used to classify stroke subtypes [5].

The neurological examination was performed on the patients and the National Institutes of Health Stroke Scale (NIHSS) was assessed at baseline, 1 hour, and 24 hours after iv-rtPA treatment. Furthermore, the NIHSS score and modified Rankin score (mRS) were obtained at 3 months to assess the long-term clinical and functional outcomes. A favorable clinical outcome was considered as mRS 0-1 and a good outcome as mRS 0–2. 

Cerebral CT scans were performed before IV rtPA bolus and repeated after 24 to 36 hours. Portable bedside TCD was performed in patients presented within 4.5 hours after symptom onset in the Emergency Department. The TCD examination was repeated one hour after the iv rtPA bolus. For the TCD examination (portable DWL), MCA (M1 branch) blood flow was detected between depths of 40–60 mm with a 2 MHz probe (right and left sides) from both temporal windows when the patient was in the supine position. All MCA (M1 branch) peak-systolic, end-diastolic, and mean flow velocity were measured. PI was also recorded on both sides. PI was calculated according to the method of Gosling [6]. Briefly, PI is calculated from the difference in the systolic and diastolic flow velocity divided by the mean flow velocity. Patients with complete arterial recanalization were diagnosed if the end-diastolic velocity improved to normal or a low-resistance stenotic signal appeared (TIBI 4 or TIBI 5). These patients were included in the analysis. 

Medical treatments given to patients after hospitalization were noted (antiplatelet, anticoagulant, antihypertensive). 

Furthermore, the patients were subjected to carotid Doppler ultrasound. The examination was done with a 7.5 MHz probe on B, C, and D-mode, respectively, when the patients were in a supine position. The common carotid arteries were examined on the bilateral and internal carotid arteries (ICA) were examined on the level, transverse and longitudinal planes (anterooblique, lateral and posterooblique). After determining the anatomic site on B-mode, bifurcation, the ICA and external carotid artery (ECA) were clearly viewed as separate. The structure of the lumen, intimal thickening, plaque, and narrowing of the lumen diameter were investigated. Bilateral flow direction and flow pattern were determined for each of the three arteries in C-mode evaluation.

The data were evaluated with the SPSS 15.0 and MedCalc statistical program. To put forward the connection of PI with the disease's different stage criteria, Pearson's correlation was used and to find the cut-off points, ROC analysis methods were used and *P* < 0.05 was considered for the statistical significance.

## 3. Results

For this study, 183 consecutive patients with acute ischemic stroke on MCA territory were examined. Twenty-five patients were excluded because of significant extracranial internal carotid artery stenosis (>70%) or occlusion. Sixty-eight patients had insufficient temporal window and were excluded from the study. The remaining of 90 of 183 patients underwent successful TCD examination during acute phase of ischemic stroke. Only 48 patients on whom complete recanalization was achieved during TCD evaluation were included in the analysis. 40% of the patients (19 patients) were female and 60% (29 patients) were male. The average age of all patients was 61.6 ± 9.8. When the patients were classified according to the etiology of ischemic stroke, 21% atherosclerosis of large vessels and 52% cardio embolism were detected, and the remaining 27% was undetermined.

When the patients' risk factors for ischemic stroke were studied, it seemed that the most common were hypertension (52%), hyperlipidemia (50%), cigarette smoking (40%), atrial fibrillation (33%), and diabetes mellitus (27%). 

IV rtPA was given within 148 minutes (range 55–245 minutes) after symptom onset. The TCD examination that was done one hour after the IV rtPA bolus demonstrated the pulsatility index on the lesion side as 1.1 ± 0.2. The MCA blood flow velocity parameters that were measured after iv rtPA bolus (in the acute stage) are given in in Table 1.

The arrival NIHSS values obtained from detailed neurological examination done on first application of patients, at 24 hours after iv rtPA treatment, and the NIHSS and mRS values obtained during the chronic period are shown in Table 2. 

**Figure 1:**
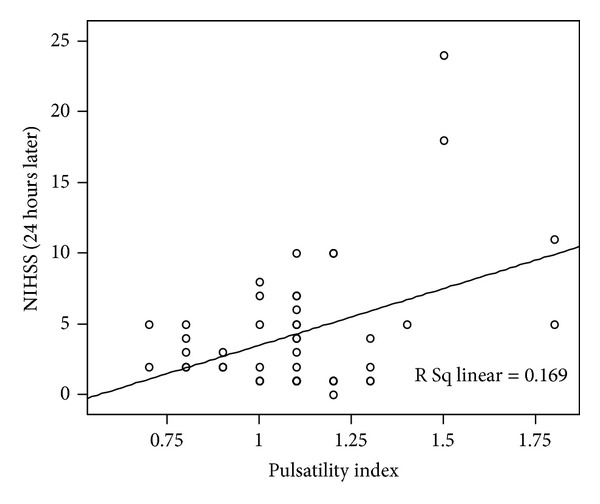
A significant correlation between the PI measured on the lesion side at the acute stage and NIHSS is observed.

**Figure 2:**
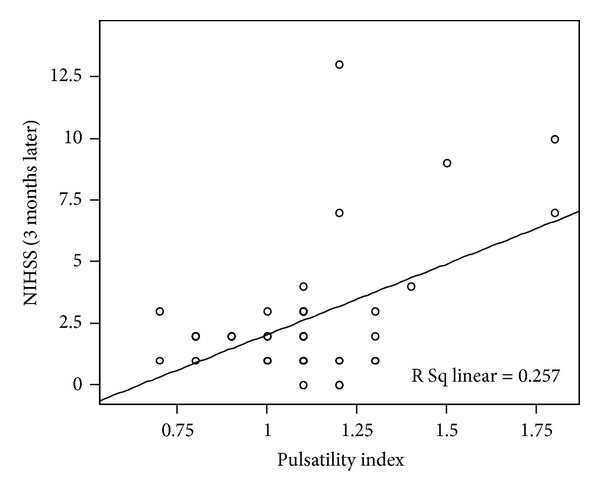
A significant correlation between the PI measured on the lesion side at the acute stage and NIHSS at 3 months is observed.

**Figure 3:**
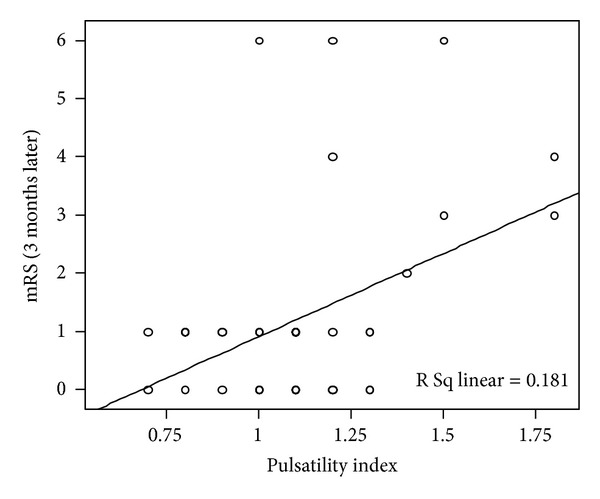
A significant correlation between the PI measured on the lesion side at the acute stage and mRS at 3 months is observed.

The pulsatility index had significant positive correlation with NIHSS values in the acute phases (Figure 1) and significant positive correlation with NIHSS (Figure 2) and mRS (Figure 3) values in the chronic phase. End-diastolic blood flow velocity had a significant negative correlation with NIHSS and mRS for every phase. Otherwise, mean and peak-systolic blood flow velocities had negative correlations with NIHSS in the acute phase and positive correlations with NIHSS and mRS values for the chronic phase; however, these correlations did not reach a significant level.

Correlations of blood flow velocity parameters on the side of the lesion with clinical scale details are given in Table 3. 

**Figure 4:**
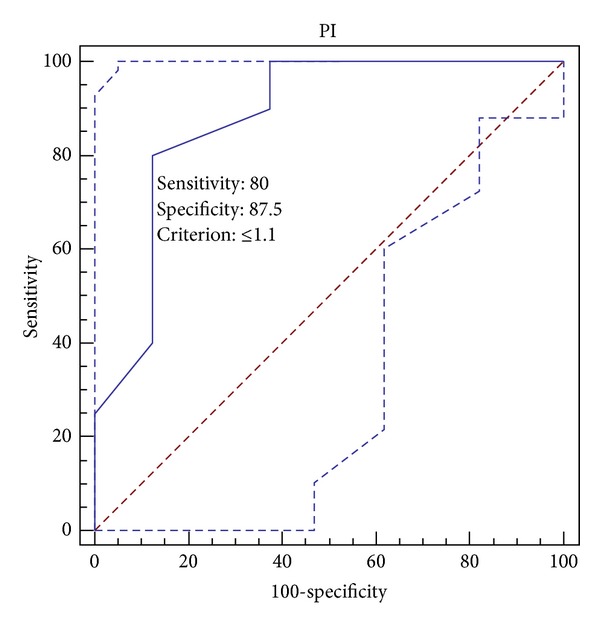
ROC curve analysis for PI and favorable outcome: area under the ROC curve (AUC): 0.878; standard error: 0.0845; 95% confidence interval: 0.751 to 0.955; *Z* statistic: 4.447; significance level *P*(Area = 0.5): 0.0001.

For a favorable outcome in the chronic phase PI might be equal to or less than 1.1 (sensitivity is 80% and specificity is 87.5%) (Figure 4).

**Figure 5:**
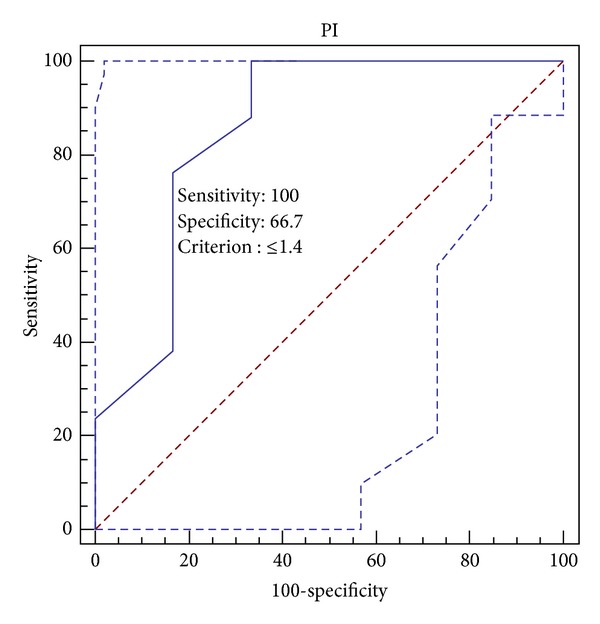
ROC curve analysis for PI and good outcome: area under the ROC curve (AUC): 0.855; standard error: 0.114; 95% confidence interval: 0.724 to 0.940; *Z* statistic: 3.111; significance level *P*(Area = 0.5): 0.0019.

For a good prognosis in the chronic phase PI might be equal to or less than 1.4 (sensitivity is 100% and specificity is 66.7%) (Figure 5).

A multivariate logistic regression analysis was performed to test whether PI is an independent predictor of poor outcome (mRS = 3–6) and admission NIHSS score, age, baseline glucose level, systolic and diastolic blood pressures, and time to treatment as well PI as values were included in the analysis. Only admission NIHSS score (*P* = 0.028) and PI (*P* < 0.001) were independently and significantly associated with poor outcome.

## 4. Discussion

TCD is a practical, noninvasive, reproducible diagnostic tool that provides indirect information about blood flow in the intracranial vessels. This technique allows the evaluation of intracranial arteries in terms of stenosis, occlusion, vasospasm, and communicating collateral flows. In previous studies, detection of an intracranial occlusion with TCD in acute ischemic stroke was regarded as a poor prognostic sign [3, 7]. Rapid neurological recovery within 24 hour after IV rtPA administration is associated with a good clinical outcome and TCD flow grades predict early neurological recovery [8, 9]. In addition, a newly published meta-analysis reveals the relationship between early treatment and TCD data and demonstrated that obstruction in the MCA is associated with poor prognosis survival which was higher in patients with a recanalized MCA [10]. Moreover, persistent arterial occlusion and reocclusion after IV rtPA are associated with poor long-term outcome and increased chance of clinical deterioration [11]. Although the majority of articles demonstrating the usefulness of TCD in the acute stroke area have focused on the effect of arterial recanalization and reocclusion on clinical and functional outcomes in response to IV rt-PA, the importance of pulsatility index has not been not emphasized [3, 7, 10, 11].

In our study, we found a negative correlation between the MCA blood flow velocities especially in end-diastolic blood flow velocities and the clinical scales that were obtained in both the acute and chronic phases of acute stroke. The association between end-diastolic blood velocities and favorable functional outcome is in parallel with a recently published study. As opposed to peak systolic velocity, a modest increase in the end-diastolic velocities is associated with favorable functional outcome and early neurological improvement [12]. However, MCA blood flow velocity measurements depend on the operator's skill and on the measurement angle as well. Hence, the pulsatility index may be a more reliable parameter than the velocity itself. 

We found a significant positive relationship between the NIHSS scores of patients at 24 hours and the PI that was measured on the recanalized MCA after iv rtPA infusion. A significant positive relationship between mRS at 3 months and PI was also found. The functional status and degree of disability for patients with higher PI values were worse. We hypothesize that an increase in the PI may indicate a poor clinical outcome even in patients with completely recanalized cerebral arteries.

PI rises when there is a barrier in measured vessel segments. Although it is most likely associated with increased intracranial pressure, increased intracranial pressure is not an expected situation during the very early stages of ischemic stroke. Some studies have shown through experiments that figural microcirculation elements of blood and fibrin play an obstructive role and that endothelium and astrocyte end-feet are swollen in ischemic cases [13]. Related to these, it was shown that segmental contraction occurs after the ischemia [14]. In addition, even though there is ischemia reperfusion in the early period, the capillary bed narrows segmentally by contraction of pericytes and the passage of blood cells is prevented [15, 16]. In a TCD study, Alexandrov et al. related the increase in end-diastolic velocity with the low resistance within the ischemic brain and the occurrence of recanalization/reperfusion [12]. In addition, we consider that the blood flow velocity measurements that were performed proximally on the vessel show higher PI in the presence of an increase in distal vascular resistance in the early period of cerebral ischemia and may reflect microcirculation problems. 

Beyond these correlations, in order to understand cut-off PI levels that give rise to more precise results in predicting good and favorable clinical and functional outcomes, ROC curve analysis was performed. A result where PI is below or equal to 1.4 indicates a good prognosis in the chronic phase whereas 1.1 and below for PI indicates a favorable prognosis in the chronic phase. In the presence of complete recanalization after iv rtPA, it is unlikely for a patient to have a worse outcome in the presence of lower PI. 

However, including only a small number of cases in this study is one of its limiting elements. We were unable to demonstrate any cut-off value for PI in order to predict a poor outcome due to the small number of patients with poor outcomes. Therefore, our preliminary findings can only be generalizable with a study including a larger sample. In addition, the cause of increased PI in patients for whom complete recanalization was achieved after iv rtPA may not be identified. Since we did not perform digital subtraction angiography in all cases, we could have missed distal embolic debris. 

In conclusion, among blood flow parameters that are measured by TCD as a simple method of examination in acute ischemic stroke, PI may play a role in predicting a good functional and clinical outcome after thrombolytic therapy in acute ischemic stroke patients. In this sense, we suggest that this examination should be carried out in all stroke units.

## Figures and Tables

**Table 1:** MCA blood flow velocity parameters on the lesion side after iv-rt-PA treatment.

	Average	Std. deviation
Mean blood flow velocity (cm/s)	38.7	9.3
Peak-systolic velocity (cm/s)	72.2	14.2
End-diastolic velocity (cm/s)	27.8	10.6
Pulsatility index	1.1	0.2

**Table 2:** Clinical scales.

Scales	Average	Std. deviation
NIHSS (before iv-rt-PA)	13.2	4.5
NIHSS (24 hours later after iv-rt-PA)	4.3	4.6
NIHSS (chronic phase)	2.6	2.6
mRS (chronic phase)	1.2	1.6

**Table 3:** Correlations of blood flow velocity parameters on the lesion side with clinical criteria.

		24 hours after NIHSS	3 months after NIHSS	3 months after mRS
Mean velocity (cm/s)	Pearson Correlation	−0.161	0.100	0.066
	Sig. (2-tailed)	0.273	0.509	0.658
	*N*	48	48	48
Peak-systolic velocity (cm/s)	Pearson Correlation	−0.278	0.109	0.056
	Sig. (2-tailed)	0.056	0.472	0.705
	*N*	48	48	48
End-diastolic velocity (cm/s)	Pearson Correlation	−0.435**	−0.317*	−0.308*
	Sig. (2-tailed)	0.002	0.032	0.033
	*N*	48	48	48
Pulsatility index	Pearson Correlation	0.411**	0.507**	0.425**
	Sig. (2-tailed)	0.004	0.000	0.003
	*N*	48	48	48

**Correlation is significant at the 0.01 level (2 tailed).

*Correlation is significant at the 0.05 level (2 tailed).
